# Cysteine-S-nitrosylation inhibits ROP5-mediated immune evasion in *Toxoplasma gondii*

**DOI:** 10.1128/msphere.00309-26

**Published:** 2026-06-30

**Authors:** S. L. Lempke, P. DiMare, B. Nichols, L. F. Fitzsimmons, A. Chaudhry, V. C. Grebe, M. L. Reese, S. E. Ewald

**Affiliations:** 1 Department of Microbiology, Immunology, and Cancer Biology at the Carter Immunology Center, University of Virginia School of Medicine 12349, Charlottesville, Virginia, USA; 2 Department of Pharmacology, University of Texas, Southwestern Medical Center 213931, Dallas, Texas, USA; 3 Department of Biochemistry, University of Texas, Southwestern Medical Center, Dallas, Texas, USA; University at Buffalo-Downtown Campus, Buffalo, New York, USA

**Keywords:** *Toxoplasma gondii*, innate immunity, cell-autonomous immunity, nitric oxide synthase, macrophages, interferons, nitrosylation, RNS, iNOS

## Abstract

Reactive nitrogen species (RNS) are a mechanism to control microbial infections conserved across the host species of the obligate intracellular parasite *Toxoplasma gondii*. Cysteine S-nitrosylation (SNO) is a reversible post-translational modification that controls complex cell behaviors by regulating protein interactions and signal transduction events. Here, we identified a cluster of *T. gondii* secreted effector proteins that are SNO-modified in a host inducible nitric oxide synthase (iNOS)-dependent manner. Among these were the rhoptry protein 5 (ROP5) paralogs, major virulence determinants in *T. gondii* and an immunodominant antigen in B6 mice. ROP5 was necessary for Type I and Type II parasites to evade IFN-γ-mediated immune clearance in iNOS-deficient macrophages. RNS led to the loss of ROP5 association with the parasitophorous vacuole membrane (PVM), which is necessary for the known functions of ROP5. Infection with ROP5 knockout parasites rescued the susceptibility of iNOS-deficient mice to infection with Type II *T. gondii*. Together, these data indicate that RNS can promote cell-autonomous parasite clearance by inhibiting the function of ROP5 alleles at the PVM.

**IMPORTANCE:**

RNS are necessary for cell-autonomous immunity to *T. gondii* infection; however, the molecular mechanisms by which RNS regulate parasite control remain poorly understood. Our findings support a model in which post-translational modification of ROP5 by RNS is a conserved mechanism of inhibiting the functions of divergent ROP5 paralogs. These data provide a specific example of how host RNS are used to counter *T. gondii* immune evasion effectors that can be applied to understand how nitrosylation regulates the function of other parasite effectors and the role of RNS in the control of other intracellular pathogens.

## INTRODUCTION

*Toxoplasma gondii* is an obligate intracellular, protozoan parasite that can infect most nucleated cells (1). The definitive hosts for *T. gondii* are feline species, which shed highly infectious oocysts (1). *T. gondii* infects a wide range of intermediate hosts, including livestock, rodents, and an estimated 30% of the human population, resulting in an infection thought to last the life of the host (2–5). Isolates from Europe and North America are predominantly one of three strains referred to as Type I, II, and III (6–9). Type II infection typically results in asymptomatic infection of immunocompetent adults and dose-dependent infection outcomes in inbred strains of mice (10). Type I is hypervirulent in many inbred strains of mice, whereas Type III requires the highest inoculum to induce sickness behavior (11). Virulence is largely determined by parasite effector proteins secreted from the rhoptry, dense granule, and microneme organelles that facilitate the development of the parasitophorous vacuole (PV), nutrient acquisition, and immune evasion.

In intermediate hosts, *T. gondii* infection occurs after ingestion of oocysts or tissue cysts contaminating food or water. *T. gondii* invades the small intestine and converts to rapidly dividing tachyzoites (1, 12, 13). The innate immune response is characterized by NF-κB signaling through activated Toll-like receptor (TLR) recognition of *T. gondii* (TLR11, TLR12), commensal bacteria (TLR2, TLR4, TLR7, TLR9), and secreted parasite effectors (GRA15, Type II) (14–19). This initiates a Th1 inflammatory cascade characterized by interleukin (IL)-1, IL-12, tumor necrosis factor (TNF), nitric oxide (NO), and interferon-gamma (IFN-γ) production (20–26). IFN-γ is required for T cell-mediated immunity to *T. gondii* and cell-autonomous control of infection (27–31).

Most murine and human cells can upregulate interferon-stimulated genes that participate in *T. gondii* control (26, 32). Two families of IFN-γ-inducible GTPases have been extensively studied in the context of *T. gondii* infection. In mouse cells, the p47 immunity-related GTPases (IRGs) and p65 guanylate-binding proteins (GBPs) oligomerize on the *T. gondii* PV, leading to vesiculation and permeabilization of the organelle (29, 33–36). In human cells, the IRGs are not functionally conserved; however, GBPs are necessary for *T. gondii* control. CIM mice originating from Southeast Asia have divergent alleles of IRGb1–2, which facilitate control of the “hypervirulent” Type I *T. gondii* (37), and several parasite effectors have evolved within species to selectively inhibit IRG proteins, underscoring the importance of this pathway for parasite control.

The parasite pseudokinase Rhoptry 5 (ROP5) is a secreted effector and the major virulence determinant in laboratory strains of mice (7, 9, 38, 39). Virulence-associated alleles of ROP5 interact with the cytosolic face of the PVM and allosterically inhibit IRGA6 oligomerization (40). In addition, IRG evasion of Type I strains is mediated by ROP5 interactions with virulent alleles of ROP-17, ROP18, and ROP39, which phosphorylate the IRG proteins and block IRG oligomerization on the PVM (41–46). Type II parasites encode a cluster of *rop5A*, *rop5B*, and *rop5C* alleles associated with lower virulence, rendering Type II parasites sensitive to successful IRG and GBP attack (37). However, the role of ROP5 in Type II infection is understudied compared to Type I. One report from the Bizik lab found that Type II strains lacking the *Rop5* locus had a small but significant increase in IRGb6 localization to the vacuole but were highly attenuated *in vivo* (10); however, IRGb6 targeting was not significantly reduced by complementation of a single copy of ROP5A or ROP5C into Pru∆*ku80*∆*rop5*. In macrophage infection, Type II ROP5 was recently implicated in the phosphorylation of TBK1 and IRF3 leading to polyubiquitination of the innate immune sensor STING when ectopically overexpressed, although the relevance of this mechanism to *in vivo* infection has not been tested (47).

Until recently, it was speculated that IRG and GBP oligomerization on the PVM was sufficient for cell-autonomous parasite killing in macrophages. However, we recently found that iNOS is necessary for efficient clearance of Type II parasites targeted by the GBPs in murine macrophages (48). *Nos2* mRNA can increase by three to four orders of magnitude in mouse macrophage and dendritic cells stimulated with NF-κB drivers and IFN-γ (49). NOS enzymes convert L-arginine into NO and citrulline (50). NO is a labile signaling molecule that reacts with strong radicals to generate reactive nitrogen species (RNS). RNS reversibly interrupt cell biological processes, rendering microbes sensitive to immune clearance (50–56). We observed that RNS inhibit *T. gondii* replication within GBP2-positive vacuoles and block egress-mediated escape from GBP2-positive vacuoles, but do not alter the percentage of GBP2-positive vacuoles (48). Moreover, PV-localized proteins are labeled with antibodies specific for nitrated tyrosine residues, an irreversible post-translational modification induced by RNS (57, 58). Tyrosine-nitration is rare and lacks robust tools for the isolation of modified proteins. In contrast, cysteine-S-nitrosylation (SNO) is a more frequent, reversible post-translational modification with established pipelines for analysis (59, 60). Although iNOS was identified as a critical mediator of *T. gondii* control in mice in the 1990s, the molecular mechanism by which RNS mediate parasite control is not known (61, 62).

In this study, we leverage tools to stabilize and purify RNS-modified proteins from *T. gondii*-infected macrophages. We identified a cluster of secreted parasite effectors that are post-translationally modified downstream of host iNOS. Among these was ROP5, the major *T. gondii* virulence effector (7, 9, 41, 45). Here, we show that SNO occurs on ROP5A, ROP5B, and ROP5C alleles. RNS lead to ROP5 dissociation from the PVM and results in enhanced clearance of Type II and Type I *T. gondii*. Finally, we show that infection with ROP5-deficient parasites trans-complements the susceptibility of iNOS-deficient mice to Type II *T. gondii* infection. Together, these data indicate that post-translational modification of ROP5 by RNS is an “Achilles’ heel” for parasite immune evasion, conserved across ROP5 alleles, that facilitates cell-autonomous control of *T. gondii* infection.

## RESULTS

### *T. gondii* secreted effectors are nitrosylated in an iNOS-dependent manner

**Fig 1:**
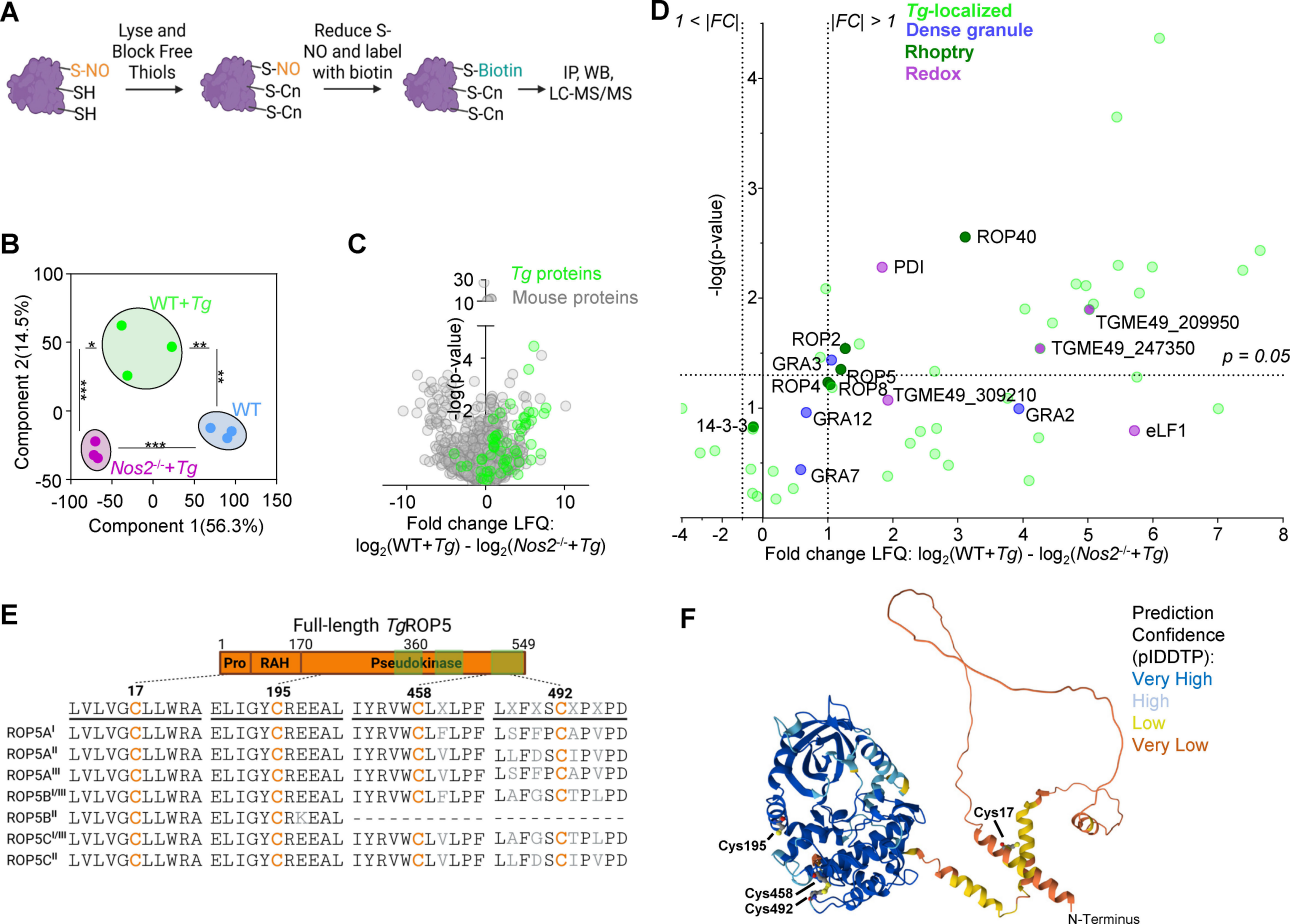
A cluster of *T. gondii* secreted effectors are nitrosylated during infection in an iNOS-dependent manner. (**A**) Schematic of SNO-biotin switch assay used to label nitrosylated mouse and *T. gondii (Tg*) proteins 6 h post-infection (hpi). (**B–D**) RAW264.7:Cas9 macrophage-like mouse cells (WT) and RAW264.7:Cas9∆Nos2 (*Nos2^−/^*^−^) were stimulated with 10 ng/mL mouse interferon-gamma (IFN-γ) and PAM3CYSK4 24 h before infection with Type II Me49gLuc at an MOI of 10. Streptavidin-precipitated mouse and *Tg* proteins were identified by LC-MS and label-free quantification. *N* = 3 biological replicate experiments. (**B**) Clustering of experimental groups by principal component analysis. Ordinary one-way ANOVA with Tukey’s multiple comparisons test was used on Component 1 (horizontal error bars) and Component 2 (vertical error bars) between groups *P < 0.05, **P < 0.005, and ***P < 0.0005. (**C and D**) Enrichment of S-nitrosylated proteins enriched in infected *Nos2^−/^*^−^ relative to infected WT samples. (**C**) Average log2 fold change of mouse (gray) and *Tg* (green) proteins. (**D**) Detailed analysis of *Tg* proteins shown in panel C. Labeled proteins are known or predicted *Tg* rhoptry (ROP, dark green), dense granule (GRA, blue), or redox-associated (purple) proteins in data set. Significance analysis represents two-tailed Student's *t*-test. Inclusion criteria for analysis required a minimum of one exclusive unique peptide per protein in two of three replicates, with an FDR of 0.05 at the peptide and protein level. (**E**) Alignment of cysteine (orange)-containing domains of *Tg*ROP5 paralogs expressed in Type I, II, and III *T. gondii*. Consensus sequence alignment to full-length *Tg*ROP5 with signal peptide/pro-domain (Pro), arginine-rich amphipathic helical (RAH) domain, and pseudokinase domain containing hypervariable variable regions (green overlay) indicated. *Tg*ROP5B^II^ is truncated at amino acid 344. Dotted lines indicate the position of cysteines within *Tg*ROP5. (**F**) Position of cysteine residues in Type II Rop5C structure predicted using AlphaFold 3 based on the Type I allele structure.

We hypothesized that RNS-mediated post-translational modifications could promote parasite killing by positively regulating host proteins involved in parasite clearance or by negatively regulating *T. gondii* secreted effectors that mediate immune evasion. To identify host and parasite proteins that are nitrosylated in an iNOS-dependent manner, we used an SNO-biotin switch assay, streptavidin precipitation, and LC-MS/MS analysis (Fig. 1A). To upregulate the transcription of GBPs and iNOS, RAW264.7 macrophage-like cell lines stably expressing Cas9 (WT) were primed with interferon-γ (IFN-γ) and the TLR2 ligand PamCys3K (PAM). Uninfected wild-type (WT) RAW, infected WT, and infected iNOS-deficient RAW (*Nos2^−/^*^−^) samples were harvested at 6 h post-infection (Fig. 1B and D). The efficiency of the SNO-biotin switch assay was validated by western blot, where few proteins were eluted from the “no-label” condition (Fig. S1A). Nitrosylation is a normal part of homeostatic cell signaling, and a similar number of proteins were isolated in each condition (Fig. S1A); however, SNO proteomes clustered by treatment condition rather than collection date using PCA analysis (Fig. 1B). Any proteins identified in the “no-label” condition were excluded from further analysis.

As expected, a subset of SNO-modified host proteins were significantly enriched in infected WT macrophages relative to uninfected macrophages (Fig. S1B gray; Table S1). This included several proteins with SNO modification sites that have been previously identified experimentally, including iNOS (63), RNF213 (64), PTPN6 (65), PLECTIN (66), and IGF2R (67). We also identified *T. gondii* proteins that were SNO-modified in an iNOS-dependent manner (Fig. 1C). In total, 54 *T. gondii* proteins were SNO-modified in the data set (Fig. S1C; Table S2), the majority of which were more abundant in WT RAW cells relative to iNOS-deficient RAWs (Fig. 1C and D, Fig. S1C). These included several *T. gondii* proteins predicted to be involved in redox signaling (PDI, eLF1, ME49_309210, ME49_209950, and ME49_247350) (68). Several proteins were also detected in a SNO-proteome study of extracellular Type I parasites (Fig. S1C, *) (69) or are homologs of host proteins regulated by nitrosylation (Fig. 1D; Fig. S1C, †) (ENO2 [70], 14-3-3 [71, 72], HSP/SHP60 [73, 74], LDH [75], RPL18A [76, 77], GAPDH [76, 78, 79], PFKII [69, 80], PDI [69, 81–83], and ribosomal proteins).

A cluster of the *T. gondii* proteins that were SNO-modified in an iNOS-dependent manner localizes to the *T. gondii* rhoptries or dense granules (Fig. 1D, dark green, blue). These proteins are known or predicted to be secreted into the PV or host cell, consistent with our hypothesis that nitrosylation promotes *T. gondii* killing by inactivating parasite proteins involved in immune evasion. Also consistent with this hypothesis, many of these secreted effectors were previously found to be dispensable for homeostatic parasite growth in fibroblasts (Fig. S1D) (84). Among the SNO-modified secreted effectors was the pseudokinase ROP5 (Fig. 1D).

The *T. gondii rop5* locus encodes ROP5A, ROP5B, and ROP5C, alleles that arose from paralogous gene expansion (Fig. 1E). Type I and Type III parasites express very similar *rop5A*, *rop5B*, and *rop5C* alleles at varying copy numbers (7, 38, 85). In comparison, Type II *rop5A*, *rop5B*, and *rop5C* alleles are divergent. *Rop5B*^II^ alleles have a premature stop codon before the hypervariable IRG-interaction domain, and Type II parasites encode a higher copy number of *rop5B^II^* and *rop5C^II^* alleles than Type I and Type III (7, 9). All *rop5* copies, across allelic type and strain, encode four conserved cysteines, except for *rop5B*^II^ due to the premature stop codon (Fig. 1E and F). In our SNO-precipitation experiment, five of the six ROP5 peptides identified by LC-MS were conserved across ROP5 paralogs; however, one peptide was exclusive to ROP5C^II^ (Table S3). Based on the role of this family as the major determinant of Type I strain hypervirulence in mice (7, 86) and the comparatively understudied role of ROP5 in Type II infection, we next explored whether RNS regulate ROP5 function (10).

### Deletion of the *rop5* locus rescues the susceptibility of *Nos2^−/^*^−^ mice to Type II *T. gondii* infection

**Fig 2:**
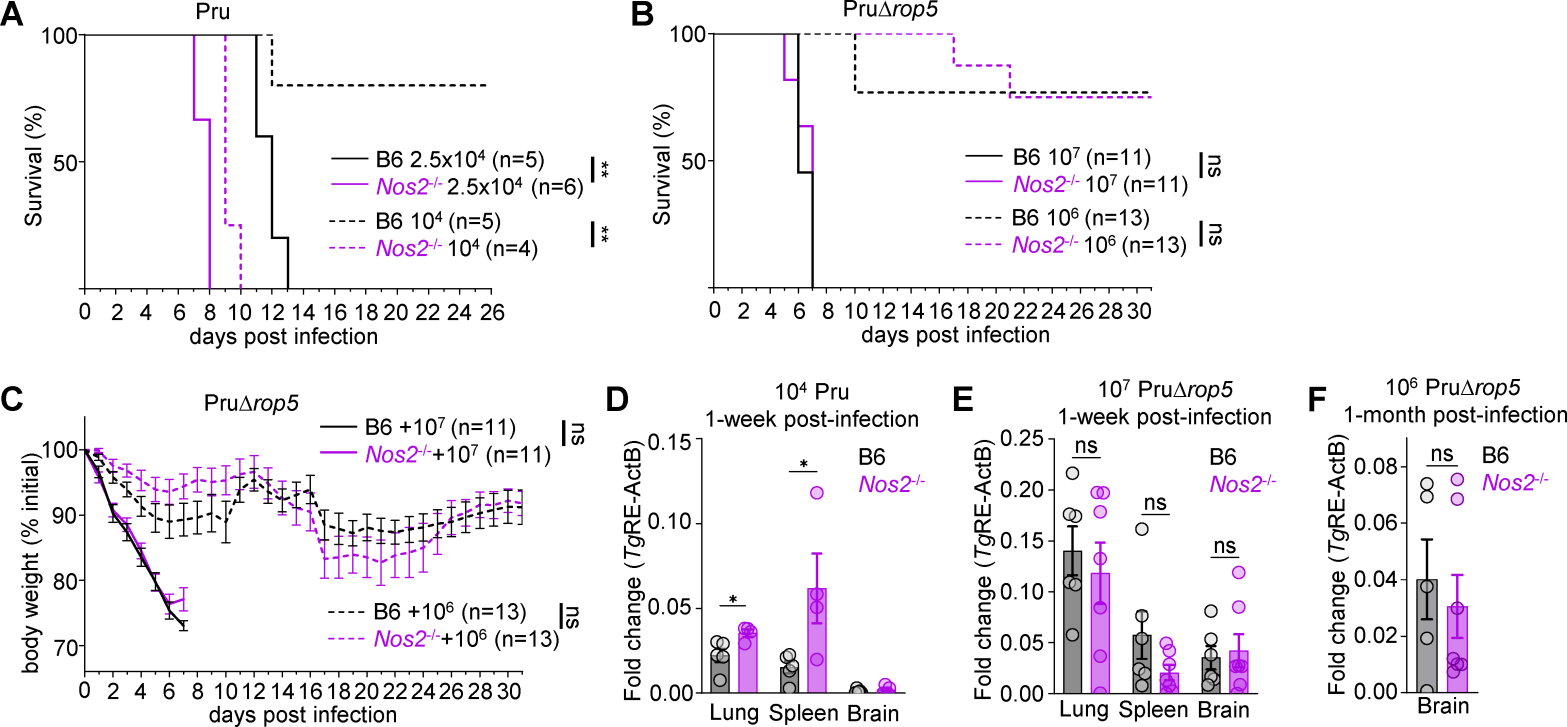
Deletion of the *Tg*ROP5 locus rescues the susceptibility of iNOS-deficient mice to *Tg* infection. B6 (WT) and *Nos2^−/^*^−^ mice were intraperitoneally (i.p.) infected with the parental Pru strain (**A and D**) and Pru∆*rop5* (**B, C, E, F**) and monitored for 31 days post-infection or until a humane endpoint was reached. (**A**) *Nos2^−/^*^−^ mice are sensitive to an inoculum of 10^4^ Pru tachyzoites, which is non-lethal to B6 mice (B6 *n* = 5, B6 *Nos2^−/^*^−^ *n* = 4), and more sensitive to an inoculum of 2.5 × 10^4^ Pru, which is lethal to B6 mice (B6 *n* = 5, *n* = 6). Each inoculum represents one independent experiment initiated on different days. (**B and C**) B6 and *Nos2^−/^*^−^ respond similarly to lethal (10^7^; B6, *Nos2^−/^*^−^, *n* = 11) and sublethal inocula (10^6^; B6, *Nos2^−/^*^−^, *n* = 13) of Pru∆ROP5 in terms of survival (**B**) and weight loss (**C**) (10^6^; B6, *Nos2^−/−^, n* = 13). Data were pooled from two independent experiments. (**D**) Pru and (**E**) Pru∆*rop5* abundance (ToxoRE) in tissues relative to mouse β-actin was measured in the lung, spleen, and brain 6–7 days post infection (10^4^ Pru B6, *n* = 5, *Nos2^−/^*^−^, *n* = 4 from one experiment, independent of infection data shown in panel A; 10^7^ Pru∆*rop5* B6, *n* = 6, *Nos2^−/^*^−^, *n* = 7 from two independent experiments) or (**F**) 10^6^ Pru∆*rop5* at 31 DPI (B6 *n* = 6, *Nos2^−/^*^−^, *n* = 7 from two independent experiments). (**A and B**) Asymmetric survival statistics with log-rank Gehan-Breslow-Wilcoxon test (ns = *P* > 0.05 and **P < 0.005) between mouse genotypes within infection dosages. (**C–F**) Parametric, unpaired *t*-test (ns = *P* > 0.05 and *P < 0.05) between genotypes.

Several independent groups have reported that mice deficient in iNOS (*Nos2^−/^*^−^) succumb to Type II *T. gondii* infections that are sublethal in wild-type mice (48, 61, 62). We recapitulated this finding using a well-tolerated infection inoculum of 10,000 Pru parasites or a lethal inoculum of 25,000 parasites (Fig. 2A). Using this background, we generated a strain lacking the *Rop5* locus (PruΔ*rop5*) (38) and confirmed the Bizik lab study showing that the *Rop5* locus is necessary for Type II virulence *in vivo* (Fig. 2A 2.5 × 10^4^ Pru vs Fig. 2B 10^7^ PruΔ*rop5*) (10). Of note, deletion of the *Rop5* locus rescued the impaired immune response of *Nos2^−/^*^−^ mice to infection, as no differences in survival (Fig. 2B) or weight loss (Fig. 2C) were observed between wild-type B6 and *Nos2^−/^*^−^ mice infected with PruΔ*rop5* at inoculum that were lethal (10^7^) or primarily sublethal (10^6^) for B6 mice. As shown previously, *Nos2^−/^*^−^ mice had higher Pru parasite load in the lung and spleen 1 week post-infection (Fig. 2D). In contrast, the PruΔ*rop5* burdens in the lung and spleen 1 week post-infection) and brain (1 month post-infection) were statistically similar between B6 and *Nos2^−/^*^−^ mice (Fig. 2E and F). In contrast, *Nos2^−/^*^−^ mice infected with Pru had a significant increase in parasite load in the lung and spleen at 1 week post-infection (Fig. 2D). Taken together, these data are consistent with a model where control of Type II infection *in vivo* requires iNOS-generated RNS to inactivate ROP5.

### The *rop5* locus is necessary for Type II and Type I *T. gondii* to evade cell-autonomous immune clearance by *Nos2^−/^*^−^ macrophages

**Fig 3:**
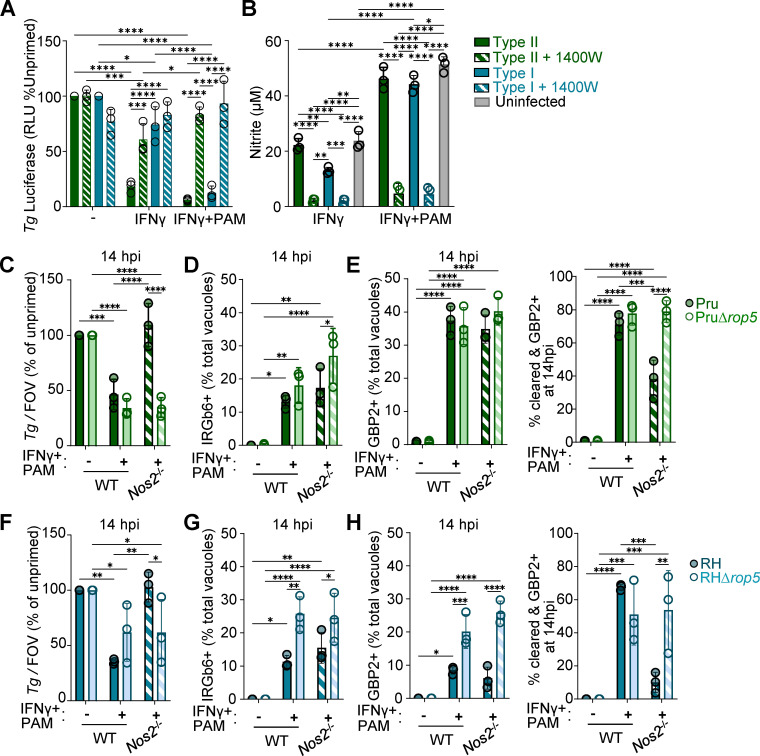
iNOS inhibits Rop5-mediated immune evasion in Type I and Type II *T. gondii*. RAWs were primed with IFN-γ with or without PAM3CSK as described in Fig. 1, followed by (**A and B**) infection with Me49-gfp-luciferase (gluc) and Type I RHgLuc with or without the iNOS inhibitor 1400W (100 μM). Twenty-four hours post-infection, parasite abundance was determined by luciferase assay (% of unprimed WT condition within strain) (**A**)**,** or nitrite levels were measured in the supernatants by Greiss assay (**B**). *N* = 3 independent experiments. (**C–H**) WT or *Nos2^−/^*^−^ RAWs were plated on coverslips, primed, and infected with Type II Pru and Pru∆*rop5* (**C–E**) or Type I RH and RH∆*rop5* (**F–H**). The mean number of parasites per field of view (α-Sag1 staining) was quantified at 14 h post-infection (**C and F**). The frequency of IRGb6-positive vacuoles was quantified per vacuole at 14 h post-infection (**D and G**). The frequency of GBP2-positive vacuoles was quantified per vacuole at 14 h post-infection (**E and H**, left panel) and used to calculate the percentage of vacuoles that had been cleared or were GBP2-positive by 14 h post-infection (**E and H**, right panel). Cumulative data is plotted from *N* = 3 independent experiments, with each dot representing results of an individual experiment. A mean of 379 vacuoles per condition was counted across three fields of view. Ordinary two-way ANOVA with Sidak’s multiple comparison within priming conditions and between strain type (*P < 0.05, **P < 0.005, ***P < 0.0005, and ****P < 0.00005).

The cysteine residues in ROP5 are conserved across strains, so we next asked if there is a role for RNS in the restriction of Type I parasites (RH). As previously reported, by 24 h post-infection, priming with IFN-γ or IFN-γ and PAM was sufficient to clear over 75% of Type II (Me49-gfp-luciferase) in a manner that was rescued by treatment with the iNOS inhibitor 1400W (Fig. 3A, green) (48). In comparison, RH-gfp-luciferase parasites were resistant to killing in IFN-γ-primed conditions (Fig. 3A, blue), consistent with the ability of RH to partially inhibit host RNS levels (Fig. 3B) (87, 88). However, when iNOS was strongly induced by IFN-γ and PAM priming (Fig. 3B), RH was efficiently cleared in a manner that depended on iNOS activity (Fig. 3A, blue).

We next asked if the iNOS-mediated control was regulated by *T. gondii* ROP5. IFN-γ and PAM significantly reduced the number of Pru and PruΔ*rop5* vacuoles observed per field of view at 14 h post-infection (Fig. 3C). The number of Pru vacuoles was rescued in *Nos2^−/^*^−^ cells. However, this was dependent on parasite expression of ROP5 (Fig. 3C, dashed bars). Impaired division of PruΔ*rop5* in *Nos2^−/^*^−^ macrophages was observed as early as 6 h post-infection (Fig. S2A) (88). Similarly, the total number of RH and RHΔ*rop5* vacuoles (Fig. 3F) and the number of dividing parasites (Fig. S2A) were reduced following IFN-γ and PAM treatment as early as 4 h post-infection. In *Nos2^−/^*^−^ RAW cells, RH vacuole number (Fig. 3F, dashed bars) and replication (Fig. S2B) were rescued by 14 h post-infection. However, the RHΔ*rop5* strain was still controlled in the absence of RNS or iNOS (Fig. 3F; Fig. S3B). Similar to the luciferase-expressing strains, we also assessed nitrite levels at 14 h post-infection and found that the RH strain was able to dampen RNS synthesis (Fig. S2C), although this was less robust than observed at 24 h of infection (Fig. 3B) and in other studies (87, 89).

In Type I infection, ROP5 alleles cooperate with ROP17, ROP18, and ROP39 to inhibit oligomerization of IRGa6, IRGb6, and IRGb10 on the PVM (41, 45, 46). Thus, we evaluated the frequency of vacuoles staining positive for IRGb6. In RAW cells treated with IFN-γ and PAM, both Pru and PruΔ*rop5* parasites had significant IRGb6 staining (Fig. 3D), and the frequency of IRGb6 on PruΔ*rop5* vacuoles was significantly enhanced in *Nos2^−/^*^−^ (Fig. 3D, hatched). A similar result was observed for Type I parasites. Notably, *Rop5*-dependent IRGB6 staining was also significantly more robust in IFN-γ- and PAM-treated WT RAWs (Fig. 3G), as expected based on the established function of Type I ROP5 paralogs (41, 45, 46).

Several reports have shown that IRG oligomerization is either upstream of or co-dependent on p65 guanylate-binding protein (GBP) recruitment to the vacuole (35, 90, 91). Thus, we evaluated the frequency of GBP2 localization to the vacuole. The frequency of GBP2 targeting was similar on Pru and PruΔ*rop5* vacuoles (Fig. 3E; Fig. S2A), whereas RHΔ*rop5* parasites had significantly more GBP2 staining at 4 (Fig. S2B) and 14 h post-infection in both WT and *Nos2^−/^*^−^ RAWs (Fig. 3H).

We previously determined that GBP2 localization is independent of iNOS (10, 37, 43, 48). However, if RNS modification inhibits ROP5 function, we would have expected higher GBP2 staining in WT RAWs compared to *Nos2^−/^*^−^ RAWs, particularly for the RH strain, and this was not observed (Fig. 3H and E left). Upon consideration of the profound difference in the total number of parental RH or Pru vacuoles in the *Nos2^−/^*^−^ (Fig. 3C and F dark dashed bars, >100% of unprimed) versus WT RAWs (Fig. 3C and F, dark bars, ~40–50% of unprimed), we reasoned that we needed a measure that would account for the vacuoles that had been targeted by the GBP/IRG system and eliminated from the culture in addition to the remaining GBP2+ vacuoles observed at the 14-hour static imaging time point (Fig. 3E and H, right). To account for this, we quantified the change in vacuole number between unprimed vs IFN-γ- and PAM-treated at 14 h post-infection (% cleared), then quantified the number of remaining vacuoles that were GBP2-positive (% GBP2+) relative to unrestrained infection in unprimed macrophages. As expected by our model that nitrosylation inhibits ROP5, primed WT RAWs had a similar frequency of parental parasites that were cleared or GBP2-positive as the Δ*rop5* strains (Fig. 3E and H right panels solid bars). By contrast, the frequency of parental strains that were cleared or GBP2-positive was significantly reduced in primed, *Nos2^−/^*^−^ RAWs (Fig. 3E and H right panels striped bars). This ability to evade GBP targeting and clearance was dependent on *rop5* (Fig. 3E and H right panels striped bars). Together, these data indicate that ROP5 is necessary for Type I and Type II to escape cell-autonomous immune clearance in a manner that is inhibited by iNOS.

### Deletion of the *rop5* locus rescues susceptibility of *Nos2^−/^*^−^ mice to Type I infection

Type I parasites are hypervirulent in B6 mice, where infection with a single parasite is a lethal dose 50 (92). To determine if RHΔ*rop5* parasites were attenuated in *Nos2^−/^*^−^ mice, similar to B6, or if *Nos2^−/^*^−^ mice were more susceptible to RHΔ*rop5* infection, we inoculated mice with a dose that was sublethal in B6 mice (10^4^) or 10^7^ tachyzoites intraperitoneally (Fig. S3A and B). We observed no differences in time to euthanasia criteria between B6 and *Nos2^−/^*^−^ (Fig. S3A) or in the kinetics of body weight loss and recovery (Fig. S3B). Further, there was no significant difference in parasite load in the brains of B6 and *Nos2^−/^*^−^ mice inoculated with 10^4^ RHΔ*rop5* tachyzoites 1 month post-infection (Fig. S3C). Cumulatively, these data indicate that *Nos2^−/^*^−^ mice are not deficient in response to RHΔ*rop5* infection, similar to what was observed with Type II strains.

### Type I ROP5A and Type I/III ROP5B are nitrosylated downstream of host iNOS

**Fig 4:**
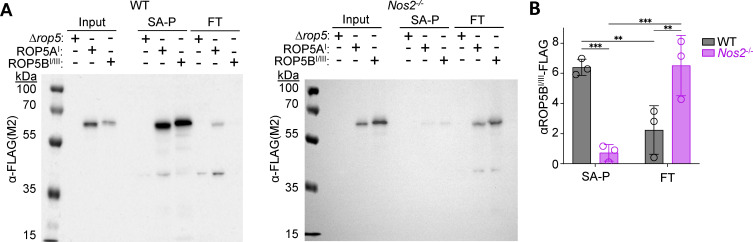
Type I ROP5A and ROP5B alleles are nitrosylated downstream of host iNOS. (**A and B**) WT and *Nos2^−/^*^−^ RAW cells were stimulated and infected with RH∆*rop5*, RH∆*rop5::rop5A^I^-HA + rop5A^I^*-FLAG (AA12, *n* = 2), and RH∆*rop5::rop5A*^I^-HA+*rop5B^I^*^/III^-3×FLAG (AC11, *n* = 3). Cells were harvested at 6 h post-infection and subjected to SNO-biotin switch assay (Input). Biotinylated proteins were isolated by streptavidin precipitation (SA-P) and collected, along with the flow through (FT), and western blotting was performed as described elsewhere. (**B**) Quantification of band intensity for *Tg*ROP5B^I/III^, shown as the band intensity relative to input in the HSS over the total band intensity in HSS and HSP combined for each protein. Ordinary two-way ANOVA with Sidak’s multiple comparison within conditions and host cell type. **P < 0.005 and ***P < 0.0005.

To determine if Type I alleles of *rop5* associated with virulence could be nitrosylated downstream of iNOS, we evaluated RHΔ*rop5* parasites complemented with two copies of the *rop5A* coding sequence from Type I (RHΔ*rop5::2x_rop5A*^I^-FLAG) or a copy of *rop5A*^I^ and the *rop5B* allele conserved in Type I and Type III parasites (RHΔ*rop5::rop5A*^I^-HA+*rop5B*^I/III^-FLAG). Both FLAG-tagged ROP5A^I^ and ROP5B^I/III^ were efficiently precipitated by SNO-biotin switch from infected RAW cells (Fig. 4A). ROP5B^I/III^ nitrosylation was significantly reduced when *Nos2^−/^*^−^ RAWs were infected (Fig. 4A and B). These data indicate that RNS may be a mechanism for broadly inactivating ROP5 proteins.

We next asked if complementation with Type I *rop5A* and *rop5B* (one copy each) was sufficient to regulate parasite survival and GBP localization. As expected, RHΔ*rop5::rop5A^I^ + rop5B^I/III^* complemented parasites did not have a significant rescue of vacuole number or parasite division at 14 hours post-infection compared to RH and RHΔ*rop5* (Fig. S4A and B). However, RHΔ*rop5::rop5A^I^ + rop5B^I/III^* survival and division in IFN-γ- and PAM-stimulated *Nos2^−/^*^−^ macrophages was significantly higher than primed, WT RAWs (Fig. S4A and B, hatched orange vs solid orange). The frequency of GBP2-positive vacuoles was also lower in primed *Nos2^−/^*^−^ compared to WT (Fig. S4C). Moreover, when the percent of parasites that were cleared or GBP2-positive at 14 h was quantified, RHΔ*rop5::rop5A^I^ + rop5B^I/III^* parasites were statistically similar to RH and different from RHΔ*rop5* strains in infected, primed RAWs and *Nos2^−/^*^−^ macrophages (Fig. S4C). Together, these data are consistent with a partial gain of resistance to IFN-γ-inducible GTPase attack.

### iNOS inhibits ROP5 association with the PVM

**Fig 5:**
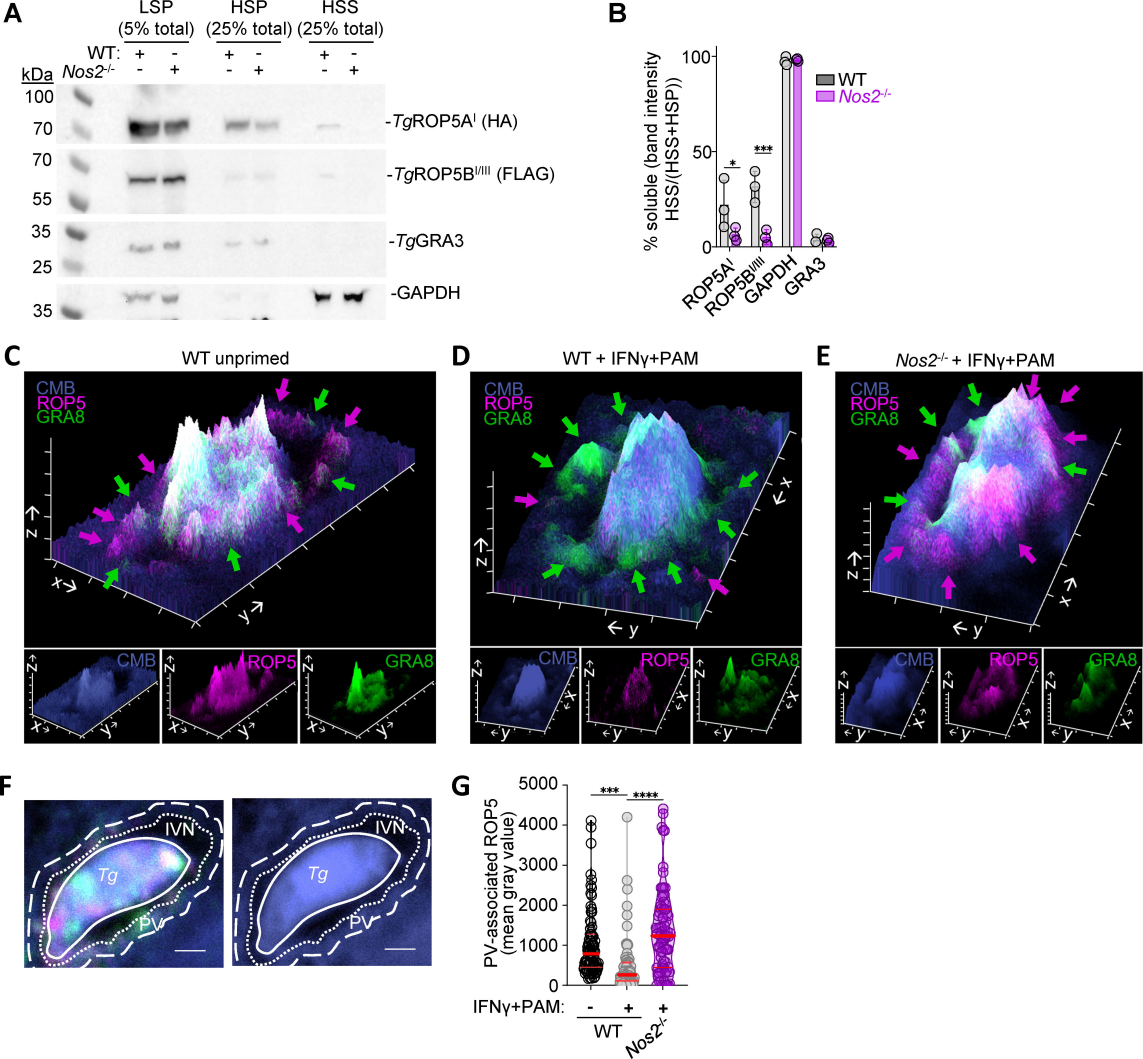
RNS inhibit ROP5 association with the parasitophorous vacuole membranes. (**A and B**) WT and *Nos2^−/^*^−^ RAW macrophages were primed as previously described and infected with RH∆*rop5::rop5AI^-HA^*AI-HA + *rop5B*^I/III^-3×FLAG at an MOI of 10. After 4 h, samples were syringe-lysed and subjected to low-speed centrifugation (LSP, 5% of sample loaded) to eliminate host nuclei and intact parasites. Supernatants were then ultracentrifuged to isolate the membranous, high-speed pellet (HSP, 25% of sample loaded), and the cytosolic, high-speed supernatant was precipitated (HSS, 25% of sample loaded). *T. gondii* ROP5B-FLAG, ROP5A-HA, GRA3 (membrane-bound effector), and host GAPDH (cytosolic protein) were detected by western blot (**A**), and the volume-adjusted band densities were plotted as the percent of each protein detected in the HSS relative to protein levels detected in the HSS plus the HSP fractions (**B**). Parametric, unpaired *t*-test; **P < 0.05 and ***P < 0.005 between genotypes. *N* = 3 biological replicate infections. (**C–G**) WT and *Nos2^−/^*^−^ RAW macrophages were plated on coverslips, primed, and infected at an MOI of 2 as described in Fig. 3. At 6 h post-infection, samples were fixed and stained with CellMask Blue, for *T. gondii* GRA8 (green), or ROP5B^I/III^ (anti-FLAG, magenta). Axis tick marks and scale bar = 1 µm. (**C–E**) Representative 2.5D reconstruction of individual parasites from 63× AiryScan images, where each z-stack = 0.64 nm. Arrows indicate secreted effector localization to the PV region. (**F and G**) CellMask Blue (CMB) stains the parasite body (Tg; solid white line) and the host cytosol, leaving a CellMask-negative region corresponding to the intravacuolar network region (IVN; between solid and dotted white lines). ROP5B^I/III^ localization to the 0.5 μm region at the edge of the cell mask boundary (“PV” between dotted and large dash lines) was quantified (**G**). Each point represents the mean gray value of ROP5B averaged across z-stack of an individual parasite. For **C–G**, *n* = 73–78 vacuoles per condition, collected in three independent experiments. Ordinary one-way ANOVA with Turkey’s multiple comparison test; ***P < 0.005 and ****P < 0.0005.

The known functions of ROP5 depend on interaction with the PVM (40), so we hypothesized that RNS could interrupt ROP5 membrane association. To assess the sub-cellular localization of ROP5, we infected IFN-γ- and PAM-primed WT or *Nos2^−/^*^−^ RAW macrophages with RHΔ*rop5::r*op5A^I^-HA + *rop*5B^I/III^-FLAG. At 6 h post-infection, samples were syringe-lysed and subjected to cellular fractionation. As expected, ROP5A and ROP5B were detected at similar levels in the low-speed pellet (LSP) of WT and *Nos2^−/^*^−^ RAW cells, which include host nuclei and intact *T. gondii* (Fig. 5A, LSP). Similar levels of ROP5A and ROP5B were also detected in the high-speed pellet (HSP) fraction, which contains host and parasite vacuole membrane-associated proteins, including *T. gondii* GRA3 (Fig. 5A, HSP). In contrast, ROP5A and ROP5B were identified in the host-cytosol, high-speed soluble (HSS) fraction of infected WT, not *Nos2^−/^*^−^ RAWs (Fig. 5A and B, mouse GAPDH). The depletion of ROP5A and ROP5B in the cytosolic fraction of *Nos2^−/^*^−^ RAWs was significant compared to WT (Fig. 5B), while GAPDH levels were similar. These data are consistent with a model where protein levels of ROP5 in the parasite are not affected by RNS; however, once ROP5 is secreted, RNS cause ROP5 to lose association with the PVM.

To test this model directly, we evaluated ROP5B^I/III^ association with the PV by immunofluorescence microscopy. CellMask is a reagent that stains the parasite and host cell, resulting in a CellMask-negative region corresponding to the intravacuolar network of the PV (36, 48). In unprimed RAW cells, ROP5 localized to the parasite and the cytosolic face of the cell mask-negative vacuole region (Fig. 5C, magenta arrows). The intravacuolar network protein GRA8 partially overlapped with ROP5 localization (Fig. 5C, green arrows). IFN-γ and PAM priming significantly reduced the localization of ROP5 to the cytosolic face of the vacuole region, but not GRA8 (Fig. 5D). ROP5 localization to the periphery of the vacuole was rescued in *Nos2^−/^*^−^ RAW cells primed with IFN-γ and PAM (Fig. 5E). To quantify ROP5 localization to the region of the PVM, the mean gray value of the ROP5 signal was quantified in a 0.5 μm region encompassing the cell mask perimeter of each vacuole image in a z-stack (Fig. 5F PV; Fig. S5A through C). This indicated that ROP5 localized to the vacuole perimeter was significantly lower in IFN-γ- and PAM-primed RAWs compared to *Nos2^−/^*^−^ RAWs and unprimed RAWs (Fig. 5H). This loss of ROP5 intensity was also observed when the entire CellMask-negative and vacuole perimeter regions were pooled (Fig. S5D). By contrast, GRA8 levels were slightly higher in IFN-γ- and PAM-primed vacuole regions compared to *Nos2^−/^*^−^ and unprimed RAWs, indicating that RNS did not trigger a global loss of secreted protein association with the vacuole (Fig. S5E). Together, these data indicate that nitrosylation of ROP5 downstream of iNOS leads to ROP5 dissociation from the PVM, rendering parasites more susceptible to cell-autonomous immune clearance.

## DISCUSSION

The antimicrobial role of reactive nitrogen species is conserved across the intermediate hosts of *T. gondii*. Our data are consistent with the conclusion that RNS can promote parasite killing by inhibiting the function of parasite effectors that mediate immune evasion. Here, we show that ROP5 proteins are nitrosylated downstream of host iNOS activity (Fig. 1C and D and 4A and B). Further, ROP5 localization to the PVM, which is essential for the known functions of ROP5, is disrupted following exposure to RNS (Fig. 5) (43). These findings are consistent with the better-studied role of RNS in the setting of bacterial pathogens, where RNS reversibly inhibit aspects of bacterial biology that facilitate immune evasion or adaptation to immunological stressors (53–56). This finding does not exclude the possibility that RNS may also positively regulate host effectors that mediate cell-autonomous parasite clearance. In our SNO-proteome study, host candidates for this function may be enriched in infected WT samples compared to uninfected (Fig. S1B) and *Nos2^−/^*^−^ samples (Fig. 1C). As S-nitrosylation is frequently mediated by transnitrosylation, or the transfer of an NO adduct from one SNO-modified protein to the free thiol of another protein, some of the nitrosylated host proteins may function to selectively modify parasite effectors identified in the study (93).

Our data indicate that SNO may be a mechanism of inhibiting ROP5 paralogs based on the conservation of cysteines across ROP5A, ROP5B, and ROP5C alleles and across strains. For Type I alleles of ROP5, our data indicate that RNS either prevent ROP5 association with the PVM or lead to ROP5 release from the PVM (Fig. 5), enhancing parasite susceptibility to IRG and GBP targeting (Fig. 3) (8, 33, 43, 45). Based on this model, we anticipated that RH-infected *Nos2^−/^*^−^ RAWs would have less IRG/GBP-targeted vacuoles than WT RAWs, and RHΔ*rop5* infected *Nos2^−/^*^−^ RAWs would have the highest frequency of GBP2-positive vacuoles (Fig. 3D and E and G and H). While there was a trend toward this pattern at 14 h post-infection, the iNOS-dependent differences were not significant, most likely due to the increased kinetics of parasite clearance downstream of IRG/GBP localization in the WT macrophages (Fig. 3C, F, E and H right panels) (33, 48). Live imaging experiments designed to evaluate the frequency of IRG/GBP targeting over time may resolve this discrepancy; however, we currently do not have *rop5* knockout parasites on a fluorescent background.

Our data are consistent with a conserved mechanism of ROP5 inactivation by RNS in Type I and Type II infection (Fig. 3D and E through G). Previous reports have shown that ROP5A^II^ cannot allosterically inhibit IRGa6 (43), and that ROP5B^II^, which has been shown to be the major virulence-determining paralog in Type I parasites (8, 37, 44), is truncated in Type II such that it cannot mediate IRG inhibition. Although Type II ROP5 knockout parasites were shown to have a small but significant increase in IRGa6 staining (10), and, in our hands, IRGb6 staining (Fig. 3D), it is difficult to reconcile this defect with the magnitude of attenuation *in vivo* (Fig. 2). These data suggest that Type II ROP5 may function via a mechanism independent of IRGs or at least the IRGs and chromosome 3 GBPs that have been studied extensively to date (34, 94). The allelic expansion and evolutionary divergence of ROP5 paralogs within Type II parasites could suggest that these proteins have additional undefined roles in immune evasion. Relatedly, it remains to be determined if RNS inactivate other rhoptry proteins via a similar mechanism to ROP5. For example, ROP2, ROP4, and ROP8 were also identified in our SNO proteome (Fig. 1D). These proteins are structurally related to ROP5, co-localize to the PVM (9), and have been co-immunoprecipitated with ROP18 (bait), ROP17, and ROP5 in Type I infection (45). Together, this suggests that ROP2, ROP4, and ROP8 are potential candidates to regulate ROP5 function in Type II parasites, where ROP5 is necessary for *T. gondii* survival in *Nos2^−/^*^−^ macrophages (Fig. 3C), and in mice (Fig. 2) (10), but appears to function largely independent of IRG (43–45) and GBP evasion (Fig. 3D and E).

One limitation of our study is that we could not identify the nitrosylated peptides using the SNO-biotin switch, streptavidin precipitation, and LC-MS approach. Unfortunately, complementary methodologies to stabilize and elute nitrosylated peptides yielded inconsistent results, most likely due to the low protein coverage of these proteomics approaches, further confounded by the low abundance of parasite proteins relative to the host proteome (95). Future studies to map the nitrosylation sites by targeted mutagenesis may be informative if the substitution of cysteine to alanine or serine does not result in a fundamental loss of ROP5 function. For example, it is possible that one or both disulfide bridge-forming cysteines are nitrosylated (Fig. 1F Cys458, Cys492). Nitrosylation could occur as the protein is secreted in the rhoptries, thereby blocking correct folding, or once ROP5 is exposed to the cytosol, leading to disruption of folding and dissociation from the vacuole. While mutagenesis may allow us to confirm that these cysteines are required, misfolding due to the disruption of the disulfide bridge would prevent testing if bypassing nitrosylation rescues ROP5 function. Alternatively, a SNO-proteome study on extracellular RH (naturally lysed out of fibroblasts) identified a peptide containing cysteine 195 (Fig. 1F), although this most likely reflects ROP5 stored in the rhoptries that has been modified by parasite-intrinsic RNS (69).

The importance of RNS in shaping host interactions with *T. gondii* is highlighted by the observation that Type I parasites can inhibit RNS (Fig. 3B; Fig. S2C). Our data recapitulate work from the DaMatta lab, which found that Type I parasites can blunt the synthesis of RNS by 30–50% in RAW and J774 macrophage-like cell lines (89). In this study, *Rop5* deletion led to lower nitrite levels compared to infection with the parental strain, although this was not significant, and RNS levels were not impacted by deletion of parasite effectors that regulate dense granule protein secretion (*asp5* and *myr1*) or inhibit STAT-dependent gene transcription (*ist*) (89). The Knoll lab found that deleting patatin-like phospholipase 1 (*TgPL1*) in Type II parasites increased the frequency of “degraded” vacuoles in macrophages activated with LPS and IFN-γ. This was reversed with NOS inhibitors or by infecting activated *Nos2^−/^*^−^ macrophages (87). PL-1 localizes to the intravacuolar network, suggesting that it may have evolved to counteract the functions of host RNS (87). *T. gondii* has 58 genes predicted to encode redox-associated proteins (including thioredoxins, glutaredoxins, and protein disulfide isomerases), several of which are necessary for parasite homeostatic functions and five of which were identified in our SNO proteome (Fig. 1D, purple) (68, 96, 97). At least two of the 58 are predicted rhoptry or dense granule proteins (not identified in the SNO proteome), suggesting that other secreted effectors may mitigate host RNS (68, 98).

The role of host versus parasite redox signaling in the regulation of the other SNO-modified parasite proteins identified in this study remains an open question. Nine of the 54 parasite proteins identified localize to the rhoptries or dense granules (Fig. S1C), and several traffic to the parasite plasma membrane (SAG1, SAG2A, SRS29C). The majority of the SNO-modified proteins function within the parasite, where accessibility to host RNS is comparatively limited by several membranes (PVM, the intravacuolar network membranes, and parasite plasma membranes), which can block or react with several classes of RNS (52, 99). The high levels of RNS produced by macrophages, coupled with permeabilization of these membranes by the IRG and GBP systems, may facilitate direct modification of proteins sequestered within the parasite by host RNS (32, 33). Additionally, host RNS may enhance parasite redox stress, thereby increasing the modification of these proteins through parasite-intrinsic pathways. Notably, 14 of the 54 proteins identified in our study were also detected in a SNO-proteome study of Type I tachyzoites that naturally lysed out of fibroblasts, which may be consistent with the latter scenario for some SNO-proteins (Fig. S1C, *) (69).

Finally, our *in vivo* data indicate that iNOS-deficient mice are more susceptible to Type II parasite infection (Fig. 2) (48, 61). In addition to its role in parasite evasion of cell-autonomous immunity, as discussed above, ROP5 is an immunodominant peptide conferring protective CD8 T cell responses in B6 mice (100). Protective CD8 responses require ERAAP to import and process cytosolic antigens in the endoplasmic reticulum, independent of the Sec22 pathway mediating cross-presentation (101, 102). Infection with parasites expressing a ROP5 allele engineered to be secreted into the host cell cytosol led to enhanced effector CD8 T cell responses and host protection (100). Thus, our data indicate that RNS may promote adaptive immune responses *in vivo* by increasing ROP5 release into the cytosol (Fig. 5) and availability for MHC I presentation to T cells. This may also contribute to the susceptibility of *Nos2^−/^*^−^ mice to infection with WT Type II parasites, in a manner that is mitigated by infection with ROP5-deficient parasites (Fig. 2), where alternative epitopes must be used to elicit a protective adaptive immune response.

## MATERIALS AND METHODS

### Mouse strains

C57BL/6 (Jax#000664) and *Nos2^−/^*^−^ (B6.129P2-*Nos2*^tm1Lau^/J, Jax#002609) mice were obtained from the Jackson Laboratory (Bar Harbor, ME).

### *T. gondii* strains

The following strains were used for this study: Me49 expressing GFP and luciferase (Me49-gluc)(103), Type I RH expressing GFP and luciferase (RH-gluc) (103), Pru∆*ku80*∆*hxgprt* (Pru parental) (104), RH∆*ku80*∆*hxgprt* (RH parental) (38), RH∆*ku80*∆*rop5*:HXGPRT (RH∆*rop5*), RH∆*rop5::rop5A^I^-HA + rop5A^I^*-3×FLAG (AA12) (AA12), and RH*∆ku80∆rop5:rop5*A^I/III^-HA+*Rop5B^III^*-3×FLAG (AC11) (38).

Pru∆*ku80*∆*rop5*:HXGPRT (Pru∆*rop5*) were generated as previously described (10, 38). CRISPR-assisted homologous recombination in the Pru∆*ku80*∆*hxgprt* background using the HXGPRT selection gene flanked by ~1,000 bp up- and downstream of the *Rop5* locus as previously described (38). Knockouts were selected using mycophenolic acid and xanthine before confirmation with PCR.

### *T. gondii in vivo* infections

Mice were challenged with the indicated inoculum of Pru, Pru∆*rop5*, or RH∆*rop5* (38) tachyzoites in 0.2 mL PBS (with Ca^2+^Mg^2+^) by intraperitoneal injection. Before infection, mice were housed on mixed bedding for 2 weeks to normalize microbiota. Animal weights and clinical scores were assessed daily during acute infections and on alternating days throughout chronic infections. Briefly, the clinical score was based on % weight loss, appearance, posture, and activity. Each parameter was scored on a three-point scale. Animals reaching a three in any parameter or a cumulative score of eight were considered to have reached a humane endpoint and euthanized immediately. The diets of all infected animals were supplemented with soft food when mild dehydration was observed for any animal in the experiment. At 6 or 7 days post-infection, brain, spleen, and lung tissues were harvested from matched B6 and *Nos2^−/^*^−^ mice for genomic DNA extraction. Brains were also harvested at 31 days post-infection to assess chronic parasite burden by PCR. Tissues were homogenized by bead beating using a Qiagen Tissuelyzer for 3 min at 25 Hz in 1 µL of UltraPure water per mg of tissue. Genomic DNA was isolated using the DNAeasy Blood and Tissue Kit (Qiagen, 69506). Relative *T. gondii* abundance was measured by quantitative PCR of *T. gondii* 529 bp repeat element (RE) and normalized to mouse beta-actin as previously described (48, 105) using TaqMan primer/probes: 529 bp RE forward, 5′-CACAGAAGGGACAGAAGTCGAA-3′ and reverse, 5′-CAGTCCTGATATCTCTCCTCCAAGA-3′; probe: 5′-CTACAGACGCGATGCC-3 (Integrated DNA Technologies); mouse β-actin: Mm02619580_g (Thermo Fisher Scientific).

### Macrophage cell line generation and maintenance

RAW264.7 macrophages stably expressing RAW264.7:Cas9 macrophage-like mouse cells (WT) and RAW264.7:Cas9∆Nos2 (*Nos2^−/^*^−^) were generated by lentiviral CRISPR/Cas9 editing as described (48). Cells were cultured in Complete DMEM (Gibco, Waltham, MA) supplemented with 10 mM HEPES (Gibco), 1 mM sodium pyruvate (Gibco), 2 mM L-glutamine (Gibco), 100 U/mL Penicillin, 100 U/mL Streptomycin (Gibco), and 10% heat-inactivated fetal bovine serum (Sigma-Aldrich, St. Louis, MO lot#22M276). Cells were passaged at 37°C and 5% CO_2_ in cell culture–treated tissue dishes. For infections, RAW cells were collected, washed with PBS, and plated for experimentation. Cells were allowed to recover overnight before cytokine stimulation.

### Macrophage infections

*T. gondii* tachyzoites were passed on confluent human foreskin fibroblasts in Complete DMEM (see above) for up to 12 serial passages. Cell culture medium containing extracellular parasites was discarded. Intracellular *T. gondii* were scraped into fresh medium and syringe-lysed by three passages through a 22-gauge blunt-end needle (Instech Laboratories). *T. gondii* were collected by centrifugation at 310 × *g* for 8 min, resuspended in fresh media, and counted on a hemocytometer.

For parasite load assays, 35,000 cells/well (1.75 × 10^5^ cells/mL) of WT-Cas9 or RAW *Nos2^−/^*^−^ macrophage were plated in a 96-well plate (Corning 3596). The next day, cells were stimulated with 10 ng/mL mIFN-γ (R&D, 485MI100CF) and/or 10 ng/mL Pam3CSK4 (Invivogen, tlrl-pms) 24 h before infection. iNOS was inhibited by 100 µM of 1400W-HCl (Selleck Chemicals, S8337) 1 h before infection. Cells were infected with an MOI of 5 of Me49-gluc or an MOI of 2 RH-gluc. After 24 h of infection, supernatant nitrite was quantified by Greiss Assay (Thermo Fisher Scientific, G7921), and relative parasite burden measured using Steady-Luc Firefly HTS Assay Kit (Biotium, 30028-L2) according to the manufacturer’s instructions. Chemiluminescence was read with the Cytation 5 plate reader (BioTek, Winooski, VT), and relative light units were normalized to unstimulated WT RAW cells for relative *T. gondii* burden.

### Immunofluorescence staining and imaging

WT and *Nos2^−/^*^−^ RAW cells were seeded onto poly-D-lysine (MP Biomedicals)-coated #1.5 thickness glass coverslips (Harvard Apparatus) in a 24-well plate at 1.5 × 10^5^ cells/mL, stimulated, and infected at an MOI of 2 as described above. At indicated experimental endpoints, the medium was aspirated, and samples were fixed with 4% PFA (Electron Microscopy Science) in PBS without Ca^2+^Mg^2+^ (PBS−/−) for 15 min at room temperature and stored in PBS at 4°C overnight. Samples were brought to room temperature and permeabilized with 0.1% Triton X-100 (Fisher) in PBS for 30 min at room temperature. After one wash with Tris-buffered saline with 0.1% Tween-20 (TBS-T), samples were blocked with 2% BSA, 2% serum from the host of secondary antibody (Jackson ImmunoResearch donkey, 017-000-001; goat, 005-000-001), and 1:200 TruStain FcX PLUS (BioLegend) in TBS-T for 45 min. Coverslips were washed with TBS-T and incubated with primary antibodies for 2.5 h at room temperature. After three washes with TBS-T, fluorescent secondary antibodies were prepared in PBS with fresh 2 μg/mL CellMask Blue (Thermo Fisher Scientific, H32720) and incubated on samples for 1 h at room temperature. After three washes with PBS−/−, coverslips were mounted with ProLong Glass Antifade Mountant (Thermo Scientific, P36984).

Primary antibodies used were as follows: α-GBP2 (1:500; Proteintech, 11854-1-AP), α-SAG1 (1:500; Thermo Fisher Scientific, MA518268), α-FLAG(M2) (1:500; Sigma-Aldrich, F1804-200UG), and α-GRA8 (1:250; BEI, NR-50269). Rabbit anti-mouse Irgb6 antiserum 141/1 was a gift from Jonathan Howard (33). Secondary antibodies used were as follows: donkey anti-rabbit IgG (H + L), highly cross-absorbed Alexa Fluor 647 (1:500; Thermo Fisher Scientific, A-31573), and goat anti-rabbit IgG (H + L) cross-absorbed Alexa Fluor 568 (1:500; Thermo Fisher Scientific, A-11011). Coverslips were imaged on the Zeiss LSM 880 (Carl Zeiss) using either 40× (Plan-Apochromat NA1.3, Oil DIC M27) or at 63× (Plan-Apochromat NA1.4, Oil DIC M27) with AiryScan.

The measure of “% cleared and GBP2+” was determined as follows. For each biological replicate, the number of SAG1-positive vacuoles in each condition were counted at 14 h post-infection. The reduction of vacuoles in the primed condition relative to unprimed was calculated as “% clearance,” where unprimed represents 0%. The number of GBP2 and SAG1 double-positive vacuoles was then quantified and normalized to the total number of vacuoles in the unprimed condition as “% GBP2+.” These two values were added to plot the total number of vacuoles that had either been eliminated in the first 14 h of infection or were present but targeted by GBP2 as the “% cleared and GBP2+ at 14 h post-infection.”

### Immunofluorescence analysis of ROP5 association with the parasitophorous vacuole

Samples were prepared as described above and imaged as z-stack images (0.84 µm) with AiryScan. After AiryScan processing (Zeiss Zen Black) into 16-bit images, individual vacuoles were identified based on the excluded CellMask Blue signal from within the vacuole, and *T. gondii* was identified by positive CellMask Blue signal. Regions of interest (PV and *T. gondii* body) were drawn manually (ImageJ). ROP5 and GRA8 signal was quantified as mean gray value within the *T. gondii* body, within the PV, and on the PV using a 0.5 µm dilation from drawn PV ROI. Data from z-slices bisecting and individual vacuole were averaged per individual vacuole. Overlapping PVs and extracellular *T. gondii* were excluded from analysis.

### SNO-biotin switch assay

WT and *Nos2^−/^*^−^ RAW cells were seeded at 2.5 × 10^6^ cells per 15 cm treated tissue culture dish 3 days before infection. Two days before infection, the medium was replaced with fresh complete DMEM containing 10% dialyzed, heat-inactivated FBS (Complete DMEM-low Biotin, Gibco) and *T. gondii* strains were passed onto confluent HFFs in Complete DMEM-low biotin. One day prior to infection, host cells were stimulated as described above. Cells were infected with an MOI of 10. At 6 h post-infection, SNO-biotin switch (Cayman Chemical) assay was performed in low light, according to the manufacturer’s instructions, with the following modification: the blocking step was extended to 1 h. The final acetone precipitation was incubated at −30°C overnight.

### Streptavidin precipitation

Biotin-switched protein pellets were resuspended in RIPA (150 mM NaCl, 5 mM EDTA, 1% NP-40, 1% SDS, 50 mM Tris-HCl, pH 8) or LC-MS/MS IP buffer (150 mM NaCl, 1%CHAPS, 1 mM EDTA, 5% glycerol) supplemented with 1× protease inhibitor (Sigma-Aldrich, 11836170001) before BCA (Thermo Scientific) protein quantification.

Magnetic beads (Thermo Scientific) were washed in low-binding tubes (Thermo Scientific, 3451) with end-over-end mixing for 5 min before use with biotin-switched samples. For 50 μL of magnetic beads, 500 μg of SNO-biotin switch proteins were added to a final volume of 500 μL. Streptavidin precipitation occurred overnight at 4°C with gentle end-over-end mixing. Beads were immobilized on a magnetic stand for 3 min. Supernatants were removed and saved as “flow through” controls before beads were gently resuspended by pipetting and washed for 5–10 minutes with end-over-end mixing containing 1× protease inhibitor.

The wash series for western blot analysis consisted of washing twice with TBS-T (TBS-0.1% Tween-20), twice with TBS-T + 500 mM NaCl, twice with TBS-T, and once with H2O + 0.5% Tween-20 (H2O). Before the final wash, samples were transferred to new low protein-binding tubes to limit carryover. For analysis by western blot, proteins were gently eluted with 100 μL 0.1 M glycine (pH 2) for 20 min at room temperature with end-over-end mixing. Eluted proteins were neutralized with 15 μL of 1 M Tris (pH 8) and prepared for western blot, as described below.

The wash series for LC-MS/MS analysis was performed as follows: twice with TBS-C (TBS-0.02% CHAPS), once with TBS-C + 500 mM NaCl, once with TBS + 500 mM NaCl, once with TBS, and once dH2O. Before the final wash, samples were transferred to new low protein-binding tubes and submitted for analysis.

### SNO-peptide identification by liquid chromatography and mass spectrometry

Samples were submitted to the University of Virginia Biomolecular Analysis Facility. Peptides were digested off streptavidin beads with trypsin and separated chromatographically with a Thermo 75 µM × 15 cm C18 EasySpray column and detected with a Thermo Orbitrap Exploris 480 mass spectrometer system with an EasySpray ion source. Mass spectra data were analyzed with Thermo Discoverer software (Version 2.5.0.400), and MaxQuantLFQ data were analyzed in Perseus (Version 2.0.3.1) using Student’s *t*-test (permutation-based FDR) (106–108). Modifications from biotin-switch assay (free thiols + 125.1 amu and biotin-labeled + 523.5 amu) were accounted for in the analysis (Cayman Chemical). Additional modifications included methionine oxidation and cysteine carbamidomethyl with a limit of five modifications per peptide. Peptide-spectrum match and protein false discovery rates were set to 0.01, and protein identification was dependent on a minimum of one unique peptide aligned to either mouse (UP000000589) or *T. gondii* (UP000001529). For inclusion in our downstream analysis, proteins required a minimum of one species-exclusive, unique peptide in 2/3 replicates.

### Cell fractionation

WT and *Nos2^−/^*^−^ RAW cells were plated into 15 cm treated tissue culture dishes, stimulated, and infected at an MOI of 10 as previously described. Four hours post-infection, infected cells were washed and scraped into cold PBS−/− supplemented with 100 µM EDTA. Samples were pelleted at 1,100 × *g*, 4°C, for 8 min before resuspension in 3 mL PBS with protease inhibitor and syringe-lysed as previously described. Low-speed pellets (LSP) were generated at 2,500 × *g*, 4°C, for 10 min, and supernatants transferred into 2.5 mL open-top thickwall polycarbonate tubes (Beckman Coulter, 349622). High-speed pellets (HSP) and high-speed supernatant (HSS) were separated using the TLA-100.3 fixed-angle rotor (Beckman Coulter) in the Optima TLX ultracentrifuge (Beckman Coulter) at 100,000 × *g* for 2 h at 4°C. HSS was acetone-precipitated at a 1:5 sample-to-acetone ratio overnight before pelleting at 4,000 × *g* for 10 min at 4°C. All samples were resuspended in RIPA buffer and shaken on ice for 1 h. Insoluble debris from lysed LSP samples was removed by centrifugation prior to sample preparation.

### Western blot

Protein samples (see above) were resuspended and boiled in lithium dodecyl sulfate (LDS) (63 mM Tris-HCl, pH 6.8) or sodium dodecyl sulfate (SDS) buffer (10 µM DTT, 0.0005% bromophenol blue, 10% glycerol, 2% SDS) at 65°C for 10 min. Samples were sonicated in a water bath three times for 30 s with 2 min on ice in between. After SDS gel electrophoresis, proteins were transferred onto PVDF membranes (Thermo Scientific, 88520) using the Trans-Blot Turbo system (Bio-Rad). Membranes were blocked with 4% biotin-free ECL Prime Blocking Reagent (Cytiva) in TBS-T for 1 h at room temperature, followed by primary antibody incubation for 2 h at room temperature or overnight at 4°C. After three 5-minute washes with TBS-T, membranes were incubated with HRP-conjugated secondary antibody for 1 h followed by three washes in TBS-T. Pierce ECL Western Blotting Substrate (Thermo Scientific) was used to visualize the HRP on the membrane, and images were acquired using the signal accumulation method on the ChemiDoc Imager (Bio-Rad). Volumetric band intensities were measured using Image Lab software (Bio-Rad, version 6.1). Antibodies used were as follows: α-HA (1:1,000; Roche, 3F10), α-FLAG (M2) (1:500; Sigma-Aldrich, F1804-200UG), α-GRA3 (1:250; BEI, NR-50269), HRP-conjugated GAPDH (1:5,000; ProteinTech, HRP-60004), Peroxidase-Streptavidin (1:7,500; Jackson ImmunoResearch, 016-030-084), and Peroxidase AffiniPure Goat Anti-Mouse IgG (1:5,000–10,000; Jackson ImmunoResearch, 115-035-002). Band densitometry was performed in Image Lab (Bio-Rad, Version 6.1)

### ROP5 cysteine modeling

Protein sequences were sourced from ToxoDB, and models were generated using AlphaFold Server powered by AlphaFold 3.

### Data processing and statistical analysis software

Data were processed in Microsoft Excel unless otherwise noted and then plotted in GraphPad Prism 10 for the indicated statistical analysis.

## SUPPLEMENTAL MATERIAL

Supplemental Figures msphere.00309-26-s0001.pdf Figures S1 to S5.

Supplemental Tables msphere.00309-26-s0002.xlsx Tables S1 to S3.

ASM does not own the copyrights to Supplemental Material that may be linked to, or accessed through, an article. The authors have granted ASM a non-exclusive, world-wide license to publish the Supplemental Material files. Please contact the corresponding author directly for reuse.
