# Supplementary material for: Cysteine-S-nitrosylation inhibits ROP5-mediated immune evasion in Toxoplasma gondii
Source: mSphere. 2026 Jun 30;11(7):e00309-26. doi: 10.1128/msphere.00309-26 (PMC13410961; doi:10.1128/msphere.00309-26)
Supplement: Supplemental Figures — Figures S1 to S5. [file msphere.00309-26-s0001.pdf]

Figure S1

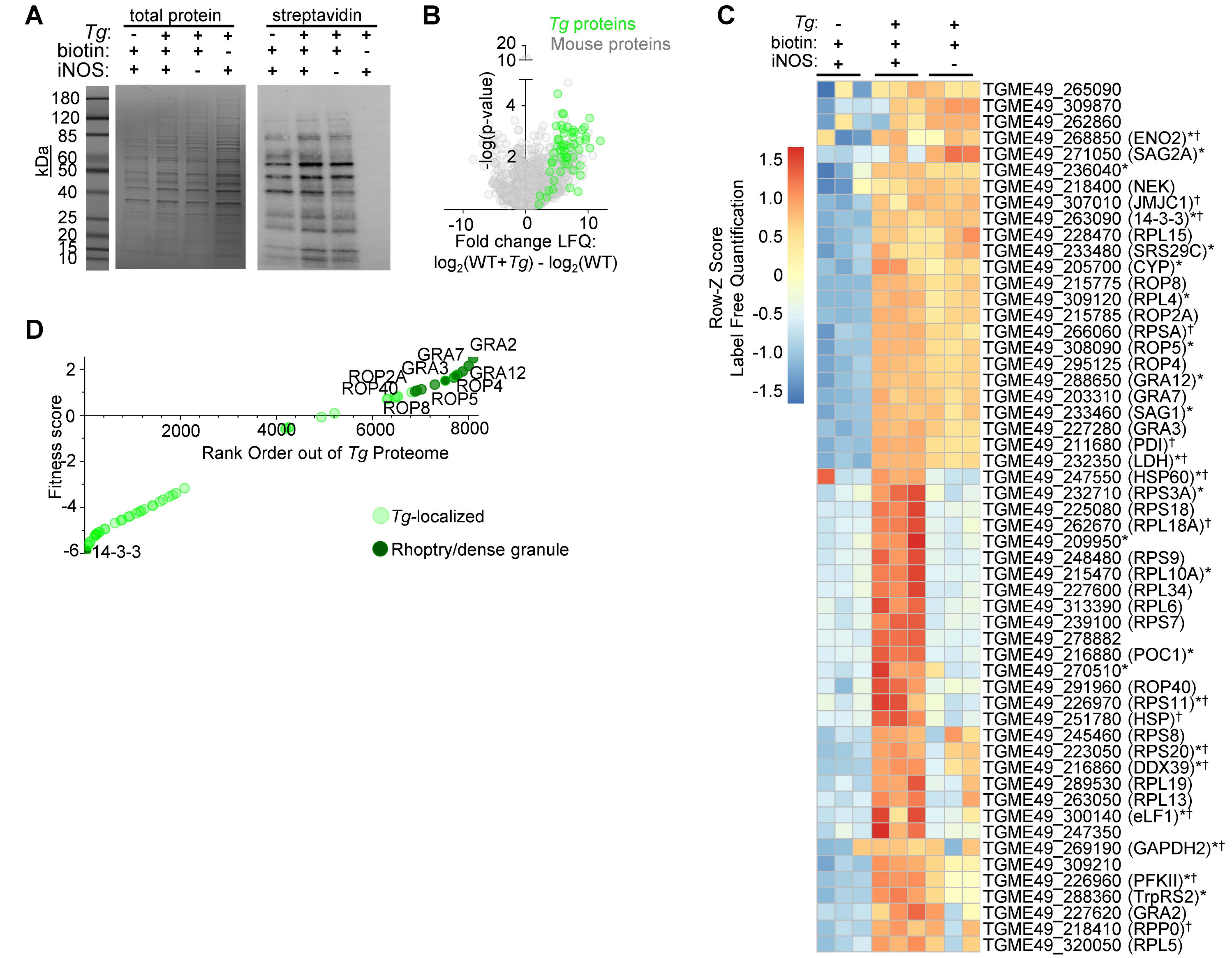

Supplemental Figure 1: Nitrosylated mouse and *T. gondii* proteins are efficiently labeled and identified by LC-MS

Nitrosylated mouse and *Tg* proteins were isolated by biotin switch assay as described in Figure 1. **A**) Representative GelCode Blue protein stain (left) and paired streptavidin blot (right) of SNO biotin switch samples isolated for LC-MS. **B**) Average log_2 fold change enrichment of S-nitrosylated proteins from uninfected WT versus infected WT RAWs. Mouse proteins (gray) and *Tg* (green) proteins. **C**) All *Tg* proteins identified across three experimental conditions are represented as row-Z score for each protein. *indicates previously identified as SNO-modified (Wang et al. *Molecules* 2023). † Indicates homologs of host proteins previously demonstrated to be nitrosylated. **D**) Nitrosylated *Tg* proteins are ranked based on dispensability during homeostatic growth (fitness score >1 is dispensable) (Lourido et al 2016). Known or predicted *Tg* rhoptry, dense granule and microneme secreted effectors are indicated in dark green.

Figure S2

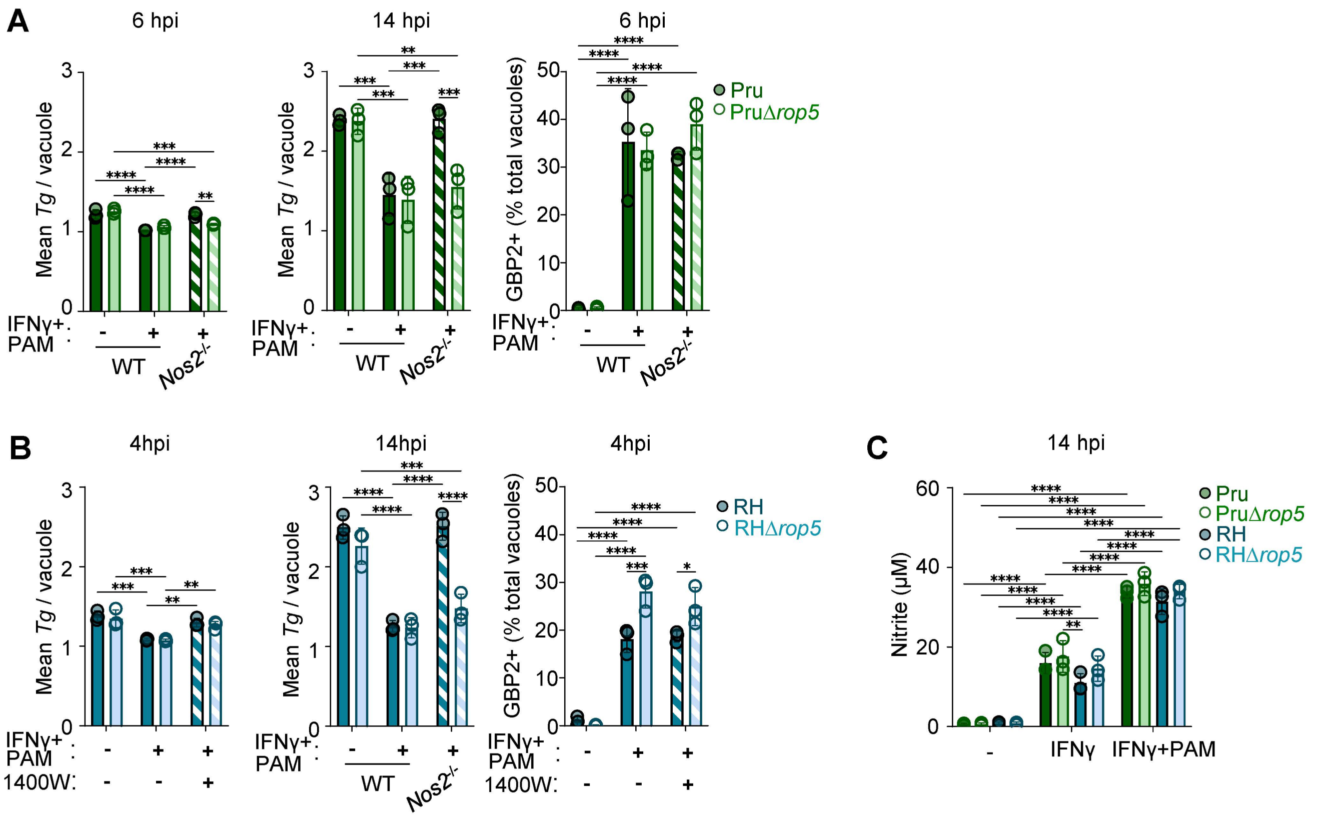

Supplemental Figure 2. iNOS inhibits Rop5-mediated immune evasion and inter-vacuole replication in Type I and Type II *T. gondii*

A-B) WT or *Nos2*$^{-/-}$ RAWs were plated on coverslips, primed with IFNγ with or without PAM3CSK as described in Figure 1 and infected with Type II Pru and PruΔ*rop5* (**A**) or Type I RH and RHΔ*rop5* (**B**). The mean number of parasites per vacuole (α-Sag1 staining) was quantified at the indicated time points (left, middle) or the frequency of GBP2-positive vacuoles was quantified (right). N=3 independent experiments. **C**) Greiss assay was performed to measure nitrite production in Type II Pru and PruΔ*rop5* or Type I RH and RHΔ*rop5* parasites at 14 hours post infection. Ordinary two-way ANOVA with Sidak's multiple comparison within priming conditions and between strain type (*= p<0.05, **= p<0.005, ***p=<0.0005, and ****p=<0.00005).

Figure S3

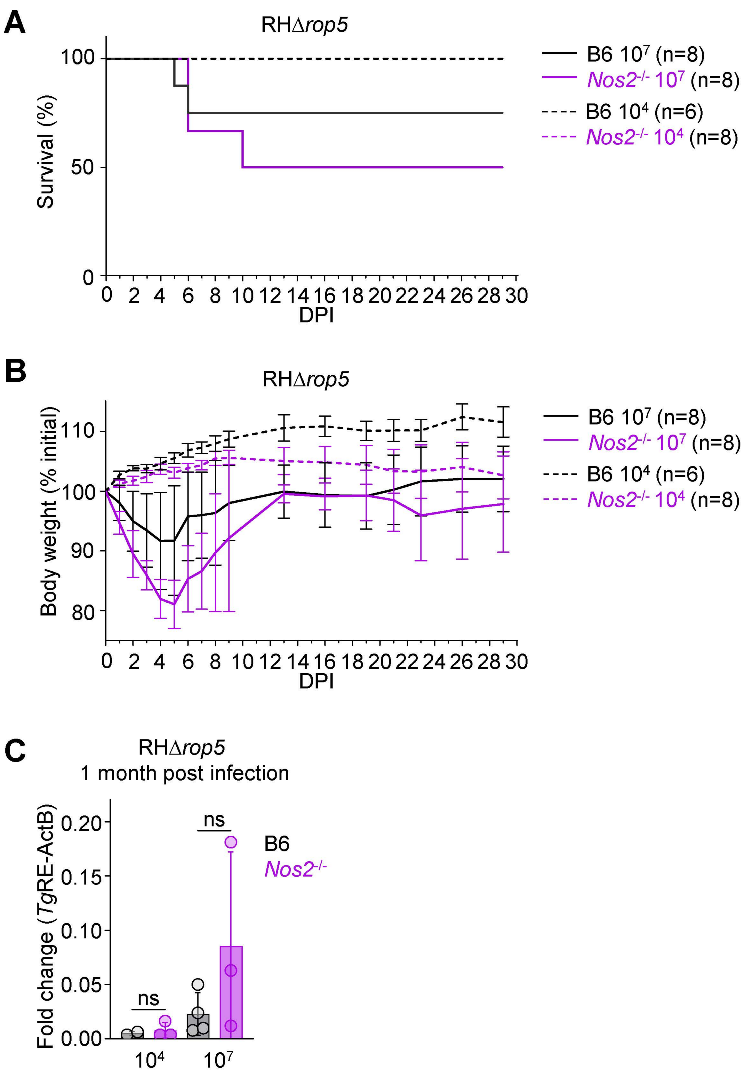

Supplemental Figure 3. Deletion of the *Tg rop5* locus rescues the susceptibility of iNOS-deficient mice to Type I *Tg* infection

B6 and *Nos2*$^{-/-}$ mice were intraperitoneally (i.p.) infected with the parental RHΔ*rop5* and monitored for 30 days post-infection or until a humane endpoint was reached. B6 and *Nos2*$^{-/-}$ B6 and *Nos2*$^{-/-}$ mice are resistant to an inoculum of 10^4 RHΔ*rop5* tachyzoites (B6 n=8, *Nos2*$^{-/-}$ n=8), and similarly sensitive to an inoculum 10^7 RHΔ*rop5* tachyzoites (B6 n=6, *Nos2*$^{-/-}$ n=8) in terms of survival (**A**) and weight loss from two independent experiments (**B**). Asymmetric survival statistics with Log rank Gehan-Breslow-Wilcoxon test (ns = p>0.05, and **p<0.005) between mouse genotypes within infection dosages. **C**) RHΔ*rop5* abundance (ToxoRE) in tissues relative to mouse was beta-Actin in the brain 30 days post infection (10^4 B6 n=2, *Nos2*$^{-/-}$ n=4; 10^7 B6 n=4, *Nos2*$^{-/-}$ n=3). Ordinary one-way ANOVA with Sidak's multiple comparisons between genotypes within dosage (ns= not significant).

Figure S4

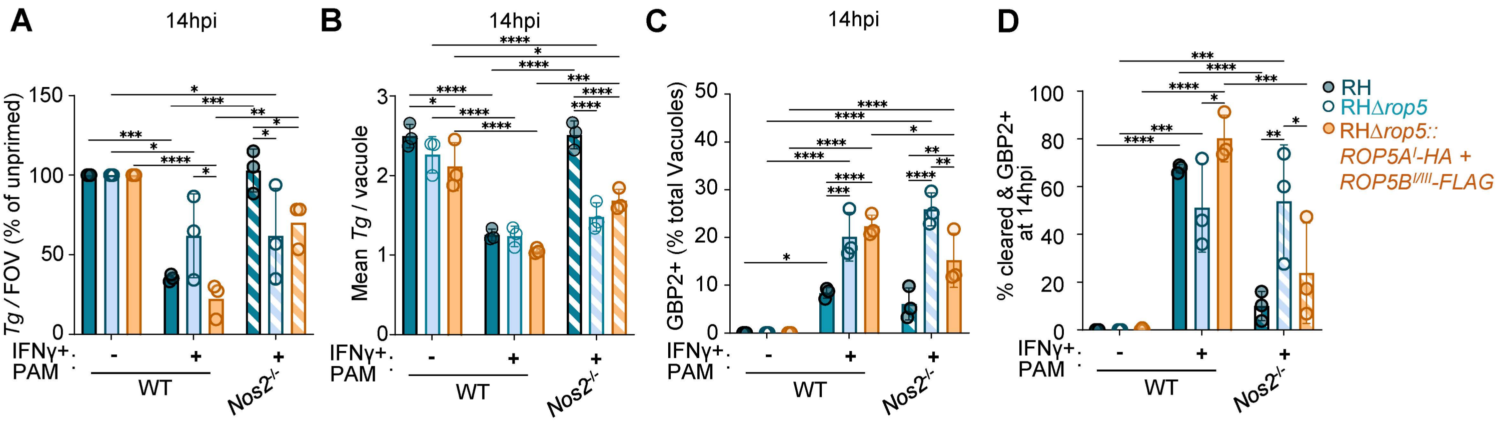

Supplemental Figure 4. Complementation of RHΔ*rop5* with *rop5A*^{I}-HA and *rop5B*$^{I/III}$-3xFLAG partially rescues resistance to GBP2 and clearance in WT versus iNOS-deficient macrophages.

A-C) WT or *Nos2*$^{-/-}$ RAWs were plated on coverslips, primed and infected with RH, RHΔ*rop5* (data shown in Figure 3F&H and Figure S2B) versus RHΔ*rop5*::*rop5A*^{I}-HA+*rop5B*$^{I/III}$-3xFLAG. The mean number of parasites vacuoles per field of view (**A**), the mean number of parasites per vacuole (**B**) the % of GBP2 positive (**C**) and the % of vacuoles cleared and GBP2 positive(**D**) at 14 hours post infection are shown. N=3 biological replicate experiments. Ordinary two-way ANOVA with Sidak's multiple comparison within priming conditions and between strain type (*= p<0.05, **= p<0.005, ***p=<0.0005, and ****p=<0.00005).

Figure S5

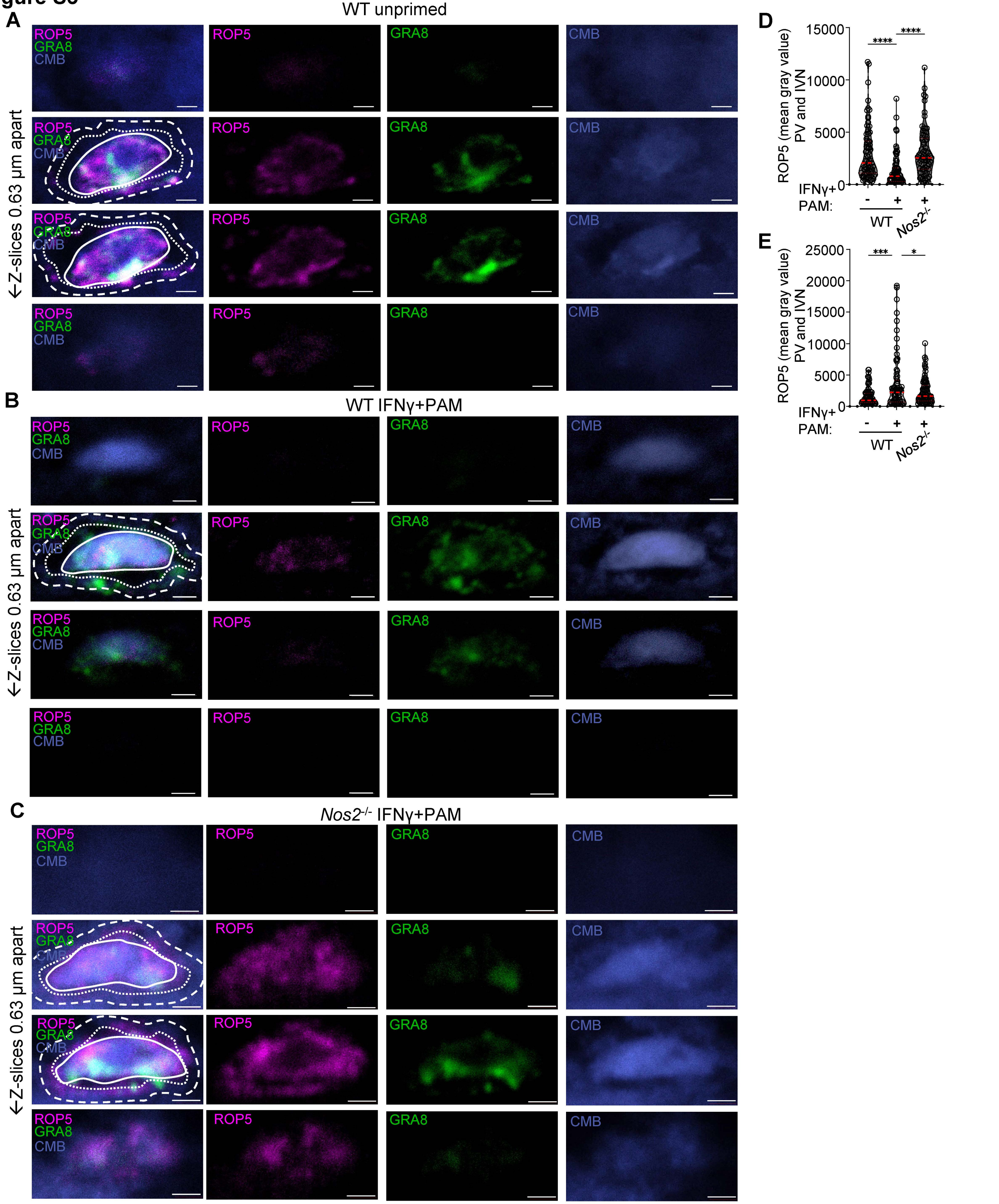

Supplemental Figure 5. ROP5B localization in representative z-stacks of parasite vacuoles

A-C) Individual z-slices from vacuoles shown in Figure 6C-E where Tg=solid white line, IVN= region between solid and dotted white lines, and PV- between dotted and large dashed lines. Outlines on merged images indicate inclusion in analysis. Scale bar = 1 µm. **D-E)** Mean grey value quantification of ROP5B (**D**) or Gra8 (**E**) in the 'vacuole' region pooled between IVN and PV regions. Each point representants the average of z-stack slices of an individual parasite vacuole, n= 73-78 vacuoles per condition, collected from three independent experiments. Ordinary one-way ANOVA with Turkey's multiple comparison test (ns = p>0.05, *** = p<0.005, and ****=p<0.0005).
